# Chyle Leak Following Radical En Bloc Esophagectomy with Two-Field Nodal Dissection: Predisposing Factors, Management, and Outcomes

**DOI:** 10.1245/s10434-020-09399-1

**Published:** 2020-12-02

**Authors:** Pamela Milito, Jakub Chmelo, Lorna Dunn, Sivesh K. Kamarajah, Anantha Madhavan, Shajahan Wahed, Arul Immanuel, S. Michael Griffin, Alexander W. Phillips

**Affiliations:** 1 Northern Oesophago-Gastric Cancer Unit, Royal Victoria Infirmary, Newcastle upon Tyne, UK; 2 University of Milan, Milan, Italy; 3 Northumbria Healthcare NHS Foundation Trust, Northumbria, UK; 4 School of Medical Education, Newcastle University, Newcastle upon Tyne, UK

## Abstract

**Background:**

Chyle leak is an uncommon complication following esophagectomy, accounting for significant morbidity and mortality; however, the optimal treatment for the chylothorax is still controversial.

**Objective:**

The aim of this study was to evaluate the incidence, management, and outcomes of chyle leaks within a specialist esophagogastric cancer center.

**Methods:**

Consecutive patients undergoing esophagectomy for esophageal cancers (adenocarcinoma or squamous cell carcinoma) between 1997 and 2017 at the Northern Oesophagogastric Unit were included from a contemporaneously maintained database. Primary outcome was overall survival, while secondary outcomes were overall complications, anastomotic leaks, and pulmonary complications.

**Results:**

During the study period, 992 patients underwent esophagectomy for esophageal cancers, and 5% (*n* = 50) of them developed chyle leaks. There was no significant difference in survival in patients who developed a chyle leak compared with those who did not (median: 40 vs. 45 months; *p* = 0.60). Patients developing chyle leaks had a significantly longer length of stay in critical care (median: 4 vs. 2 days; *p* = 0.002), but no difference in total length of hospital stay.

**Conclusion:**

Chyle leak remains a complication following esophagectomy, with limited understanding on its pathophysiology in postoperative recovery. However, these data indicate chyle leak does not have a long-term impact on patients and does not affect long-term survival.

Chyle leak after transthoracic esophagectomy has a reported incidence of 1–9%.^1^,^2^ This complication has been associated with a higher rate of morbidity and mortality among patients who underwent surgery, ranging between 0 and 50%.^1^,^3^,^4^ The reasons for this are explained by the continuous loss of chylous fluid, which is naturally rich in fats, fat-soluble vitamins, enzymes and lymphocytes, leading to a decrease in serum levels of albumin and a significant reduction in peripheral lymphocytes that can result in malnutrition and immunosuppression. Moreover, this fluid loss may lead to hypovolemia and subsequent respiratory failure.^5^,^6^

Postoperative chyle leaks result mainly from damage to, or a failure to ligate, the main thoracic duct or its tributaries during both the abdominal and thoracic phase of esophagectomy. The duct is closely related to the esophagus, but because the anatomy is highly variable, its susceptibility to be damaged is easily explained, especially when extensive nodal dissection is undertaken. In addition, chyle leak could result from damage to the cisterna chyli, during nodal dissection around the celiac trunk. In this case, the presentation may be either as chylous ascites or as chylothorax, as chyle spreads into the chest through a widened hiatus promoted by the negative pressure in the thorax.^7^

The most common presentation of chylothorax is excessive drainage via the chest tubes in the early postoperative period, with the classic cream-colored effluent seen only when the patient starts to be fed enterally or orally. The diagnosis can be confirmed by a triglyceride level of > 110 mg/dL or the presence of chylomicrons in the effluent. The optimal treatment for the chylothorax is still controversial, however both surgical and conservative management has been advocated.^8^–^11^ The aim of this study was to evaluate the incidence, management, and outcomes of chyle leaks within a dedicated esophagogastric cancer center.

## Methods

### Study Population

Data from all patients who underwent elective two-phase transthoracic esophagectomy at a dedicated esophagogastric cancer unit between January 1997 and December 2017 were extracted from a contemporaneously maintained database. The study was performed in accordance with the Declaration of Helsinki, and the study protocol was approved by the Institutional Review Board.

Data included patient demographics, tumor characteristics, tumor regression grade according to the Mandard Classification,^12^ histopathologic parameters, type of surgery, length of hospital stay, postoperative morbidity and complications, and in-hospital and 30-day mortality. Patients who received a transthoracic resection with cervical reconstruction or colonic interposition, as well as those patients found to have non-resectable disease, were excluded.

Chyle leak was diagnosed clinically by the appearance of the drain output and/or the presence of excessive (> 500 mL/24 h) drainage of chylous fluid via the intercostal or abdominal drains. Triglyceride levels and the presence of chylomicrons in the effluent were not routinely measured in this unit, but were used in incidences of uncertainty.

### Surgery

Surgery was performed in a standardized fashion as previously described, with an open laparotomy for gastric mobilization, and insertion of feeding jejunostomy and pyloroplasty. Thoracic access was obtained using a right thoracotomy. The thoracic duct was actively sought, ligated and excised as part of an en bloc lymphadenectomy. Right-sided apical and basal thoracic drains were inserted as standard.^13^ Patients were routinely cared for on the critical care unit (CCU) overnight and, providing their clinical condition allowed, returned to the ward the following day. Standard practice was to leave drains in situ until postoperative day 5 or 6. The postoperative feeding practice varied, before starting an Enhanced Recovery After Surgery (ERAS) protocol, in accordance with the operating surgeon. Since 2015, the postoperative feeding practice has followed the ERAS protocol, which involved commencement of feeding via the jejunostomy on the first postoperative day and gradually increasing over the following week.

### Chyle Leak Treatment

Once a chyle leak was diagnosed, the initial management was conservative and included strict fluid and electrolyte maintenance, in order to maintain a balance between the volume of chyle draining and the needs of the patient. Nutritional status was maintained using a medium-chain triglyceride (MCT) enteral feed or, rarely, total parenteral nutrition (TPN) if necessary. It was normal practice to start cotrimoxazole if the serum lymphocyte count was < 1000/µL. This practice was adopted due to a patient developing *Pneumocystis carinii* pneumonia prior to the time frame evaluated.^14^ The indication for returning to theater was an excess of 1000 mL/24 h, persisting and not diminishing for > 48 h despite maximum conservative therapy.

### Statistical Analysis

Categorical variables were compared using the Chi square test. Non-normally distributed data were reported as median and interquartile range (IQR) and were analyzed using the Mann–Whitney U test. Survival was estimated using Kaplan–Meier survival curves and compared using the log-rank test. A multivariable Cox regression model was developed to model clinically relevant variables predictive of long-term survival. The multivariable analysis included those variables deemed potentially clinically relevant. A *p* value < 0.05 was considered statistically significant. Data analysis was performed using R Foundation Statistical software (R 3.2.2) with TableOne, ggplot2, Hmisc, and survival packages (The R Foundation for Statistical Computing, Vienna, Austria), as previously described.^15^,^16^

## Results

### Baseline Demographics

Between January 1997 and December 2017, among 992 patients who underwent right two-stage transthoracic esophagectomy and two-field nodal dissection with curative intent, 5% (*n* = 50) developed chyle leaks. Baseline demographics of the entire cohort are presented in Table 1. There were no significant differences in age, sex, or underlying histology in patients developing chyle leak; however, those developing chyle leak had a statistically significant lower body mass index (BMI) than those without a chyle leak (median 24 vs. 26; *p* = 0.001). There were also no significant differences in American Society of Anesthesiologists (ASA) grade or treatment received (i.e. surgery vs. neoadjuvant therapy and surgery) between the two groups.

**Table 1:** Baseline demographic characteristics of the two groups of patients

	No chyle leak [*n* = 942]	Chyle leak [*n* = 50]	*p* value
Age at presentation (median [IQR])	65 [59–71]	68 [56–70]	0.839
Male	721 (77)	33 (66)	0.126
Squamocellular carcinoma	179 (19)	12 (24)	0.491
BMI (median [IQR])	26 [24–30]	24 [22–26]	0.001
Smoking status			0.206
Current	217 (23)	9 (18)	
Ex-smoker	448 (48)	20 (40)	
Never	268 (28)	21 (42)	
Unknown	9 (1)	0 (0)	
ASA grade			0.791
1	125 (13)	8 (16)	
2	509 (54)	26 (52)	
3	253 (27)	13 (26)	
4	6 (1)	1 (2)	
Unknown	49 (5)	2 (4)	
Surgery only	395 (42)	20 (40)	0.902
Neoadjuvant regimen			0.935
None	395 (42)	20 (40)	
CF	199 (21)	10 (20)	
CROSS protocol^17^	22 (2)	2 (4)	
ECF/ECX	283 (30)	15 (30)	
Unknown	43 (5)	3 (6)	
Stage of disease			0.344
0	40 (4)	3 (6)	
I	87 (9)	1 (2)	
IIa	5 (1)	1 (2)	
IIb	48 (5)	3 (6)	
III	602 (44)	31 (62)	
IV	160 (17)	11 (22)	
Tumor differentiation grade			0.057
Well	80 (8)	2 (4)	
Moderate	452 (48)	27 (54)	
Poor	354 (38)	14 (28)	
Unknown	56 (6)	7 (14)	
Number of lymph nodes (median [IQR])	31 [24–39]	32 [25–43]	0.237
Margin status R1	19 (2)	0 (0)	0.628
Lymph node involvement	430 (46)	24 (48)	0.857
Vascular involvement	330 (35)	11 (22)	0.082
Perineural involvement	418 (44)	18 (36)	0.309
Tumor regression grade			0.020
1	37 (4)	2 (4)	
2	30 (3)	2 (4)	
3	73 (8)	11 (22)	
4	168 (18)	7 (14)	
5	41 (4)	3 (6)	
Unknown	593 (63)	25 (50)	
Extracapsular spread	175 (19)	11 (22)	0.676
Critical care stay (median [IQR])	2 [1–5]	4 [–7]	0.002
Length of hospital stay (median [IQR])	15 [12–22]	19 [13–27]	0.064
Overall complications	613 (65)	49 (98)	< 0.001
SSI	101 (11)	2 (4)	0.200
Pulmonary	120 (13)	8 (16)	0.650
Cardiac	78 (8)	2 (4)	0.414
Anastomotic leak	75 (8)	5 (10)	0.803
In-hospital mortality	28 (3)	3 (6)	0.434
30-day mortality	22 (2)	0 (0)	0.548

Data are expressed as *n* (%) unless otherwise specified

*ASA* American Society of Anesthesiologists, *BMI* body mass index, *CF* cisplatin/5-fluorouracil, *CROSS protocol* chemoradiotherapy for oesophageal cancer followed by surgery study, *ECF/ECX* epirubicin, cisplatin plus either 5-fluorouracil or capecitabine, *IQR* interquartile range, *SSI* surgical site infection

### Postoperative Outcomes

Patients developing a chyle leak had a significantly longer length of critical care stay (median: 4 vs. 2 days; *p* = 0.002) but no significant differences in overall length of hospital stay. There were no significant differences in rates of other complications between those with and without a chyle leak (Table 1). Furthermore, there were no significant differences in in-hospital (6% vs. 3%; *p* = 0.434) and 30-day (0% vs. 2%; *p* = 0.548) mortality in patients with and without a chyle leak.

### Overall Survival and Prognostic Factors

**Fig. 1:**
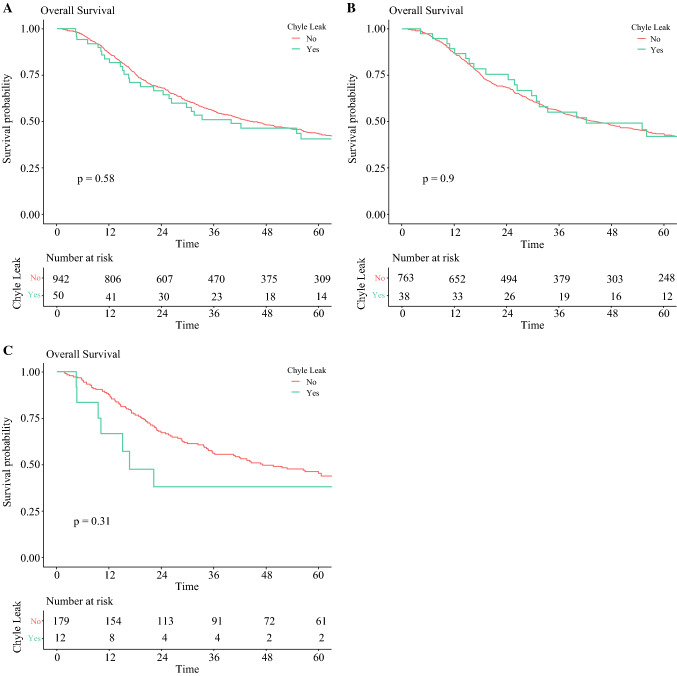
Overall survival rates for patients undergoing esophagectomy developing chyle leak versus **a** no chyle leak, **b** no chyle leak in the subgroup of patients with adenocarcinoma, and **c** no chyle leak in the subgroup of patients with squamous cell carcinoma

The overall survival of patients is presented in Fig. 1. There was no significant difference in overall survival between patients without a chyle leak and those with a leak (median: 40 vs. 45 months; *p* = 0.58). Furthermore, in subgroup analysis by underlying tumor histology, there were no significant differences in survival in patients with and without a chyle leak for adenocarcinoma (median: 42 vs. 44 months; *p* = 0.90) and squamous cell carcinoma (median: 17 vs. 47 months; *p* = 0.31) (Table 2).

**Table 2:** Median overall survival in all patients, and in patients affected by adenocarcinoma and squamous cell carcinoma according to whether there was No chyle leak or a chyle leak occurred (Yes)

	No. of patients	Median overall survival, months	*p* value
*All patients*			
No	942	44.5 (40.1–52.6)	0.58
Yes	50	40.0 (25.8–NR)	
*Adenocarcinoma*			
No	763	44.3 (38.5–52.6)	0.90
Yes	38	42.2 (29.7–NR)	
*Squamous cell carcinoma*			
No	179	47.2 (35.6–68.8)	0.31
Yes	12	16.7 (10.1–NR)	

*NR* not reached

On univariable analysis, age, poor and moderate tumor grade, positive margin status, positive lymphatic, vascular and perineural invasion, extracapsular spread, tumor regression grade 5, and stage III and IV disease were independent prognostic factors for long-term survival. Chyle leak was not a prognostic factor of survival on either univariable or multivariable analysis (hazard ratio [HR] 0.99, 95% confidence interval [CI_95 %_] 0.67–1.45, *p* = 0.949; HR 1.09, CI_95 %_ 0.73–1.63, *p* = 0.675).

On multivariable analysis, age, male sex, squamous cell carcinoma, year of operation, poor tumor grade, margin status, lymph node and perineural involvement, and extracapsular spread were identified as prognostic factors for long-term survival (Table 3).

**Table 3:** Results of univariable and multivariable analysis for overall survival

	Univariable		Multivariable	
	HR (95% CI)	*p* value	HR (95% CI)	*p* value
*Age at presentation*				
Mean	1.02 (1.01–1.03)	< 0.001	1.02 (1.01–1.03)	< 0.001
*Sex*				
Male	Ref		Ref	
Female	0.84 (0.69–1.01)	0.066	0.73 (0.59–0.90)	0.003
*ASA grade*				
1	Ref		Ref	
2	1.15 (0.90–1.47)	0.260	1.11 (0.86–1.43)	0.421
3	1.56 (1.20–2.03)	0.001	1.27 (0.97–1.66)	0.088
4	1.60 (0.65–3.95)	0.309	1.48 (0.57–3.80)	0.421
Unknown	0.89 (0.59–1.36)	0.595	0.76 (0.50–1.17)	0.212
*Histology*				
Adenocarcinoma	1.07 (0.44–2.57)	0.888	0.54 (0.22–1.32)	0.178
Others	0.52 (0.20–1.39)	0.192	0.54 (0.20–1.45)	0.220
SCC	0.96 (0.39–2.34)	0.921	0.66 (0.26–1.66)	0.379
*Operation year*				
	0.99 (0.97–1.00)	0.155	0.97 (0.95–1.00)	0.031
*Treatment*				
Surgery only	Ref		Ref	
NAC + surgery	1.01 (0.86–1.18)	0.925	0.87 (0.71–1.08)	0.208
*Tumor grade*				
Well	Ref		Ref	
Moderate	1.96 (1.39–2.75)	< 0.001	1.37 (0.95–1.97)	0.091
Poor	2.92 (2.07–4.11)	< 0.001	1.65 (1.12–2.41)	0.011
Unknown	1.24 (0.75–2.04)	0.400	1.84 (1.03–3.31)	0.040
*Margin status*				
R0	Ref		Ref	
R1	5.85 (3.67–9.33)	< 0.001	2.59 (1.58–4.23)	< 0.001
*Lymphatic invasion*				
No	Ref		Ref	
Yes	2.37 (2.01–2.78)	< 0.001	1.33 (1.08–1.64)	0.008
*Vascular invasion*				
No	Ref		Ref	
Yes	2.08 (1.77–2.45)	< 0.001	0.92 (0.75–1.13)	0.415
*Perineural invasion*				
No	Ref		Ref	
Yes	2.56 (2.18–3.00)	< 0.001	1.36 (1.11–1.67)	0.003
*Tumor regression grade*				
1	Ref		Ref	
2	0.96 (0.40–2.31)	0.926	0.8 (0.32–1.99)	0.632
3	1.38 (0.70–2.74)	0.354	0.92 (0.45–1.88)	0.812
4	2.92 (1.57–5.44)	0.001	1.2 (0.62–2.34)	0.588
5	4.01 (2.04–7.89)	< 0.001	1.35 (0.65–2.80)	0.418
Unknown	2.22 (1.22–4.05)	0.009	1.28 (0.68–2.41)	0.448
*Extracapsular spread*				
No	Ref		Ref	
Yes	3.06 (2.54–3.69)	< 0.001	1.89 (1.48–2.40)	< 0.001
*pStage*				
0	Ref		Ref	
I	1.30 (0.74–2.28)	0.357	1.41 (0.72–2.76)	0.317
II	1.55 (0.89–2.71)	0.125	1.32 (0.68–2.58)	0.411
III	4.67 (2.74–7.98)	< 0.001	3.04 (1.57–5.88)	0.001
IV	8.37 (4.72–14.82)	< 0.001	4.24 (2.09–8.59)	< 0.001
*Chyle leak*				
No	Ref		Ref	
Yes	0.99 (0.67–1.45)	0.949	1.09 (0.73–1.63)	0.675

*ASA* American Society of Anesthesiologists, *CI* confidence interval, *HR* hazard ratio, *SCC* squamous cell carcinoma, *NAC* neoadjuvant chemotherapy

### Management of Chyle Leak

Among patients who developed a chyle leak, 52% (*n* = 26) underwent surgical intervention. Identification and ligation of the thoracic duct was successful in 88.4% (*n* = 23) of patients. Two patients had a leak from both the thoracic duct and the cysterna chili, which required laparotomy and thoracotomy. One patient had a left chylothorax and another had a bilateral chylothorax. Median time to surgery was 6 days (range 2–11), median time in the CCU was 6 days (range 2–20), and median length of hospital stay was 24 days (range 9–83). Three patients required a third-look for persistent chyle leak.

**Fig. 2:**
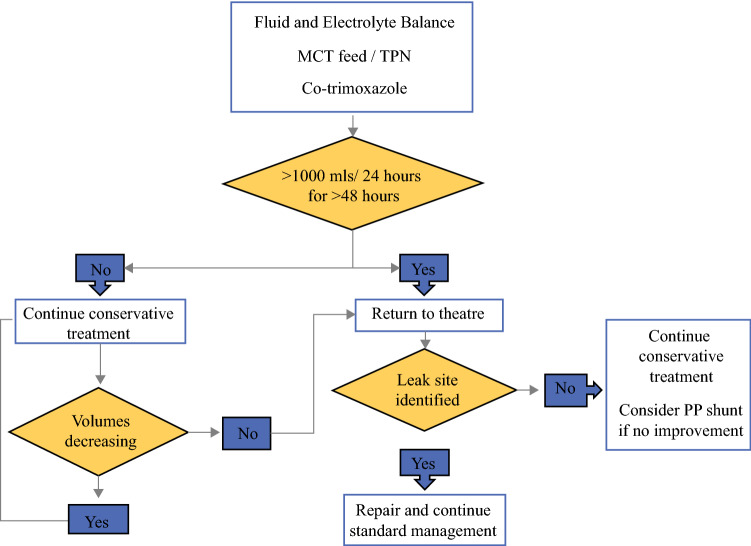
Algorithm for the management of chyle leaks following esophagectomy. *MCT* medium-chain triglyceride, *TPN* total parenteral nutrition, *PP* pleuro-peritoneal

Non-surgical management was used on 48% (*n* = 24) of patients. Two patients required pleuro-peritoneal shunt insertion, while the remaining 22 patients received aggressive, conservative treatment with maintenance of the pleural drain until daily drainage was < 150 mL/day. Median hospital stay for these patients was 15 days (range 7–55). Parenteral or MCT feeding was adopted depending on the chyle leak daily output and the general condition of the patient. Decisions to use shunts were made on an individual patient basis; one patient had previously undergone unsuccessful attempts to ligate the duct at surgical intervention, while the other patient had a shunt inserted and then required a thoracotomy for bleeding. Early in this series, one patient developed *Pneumocystis carinii*-related pneumonia and required treatment with cotrimoxazole. It has subsequently become standard practice to commence cotrimoxazole once a chyle leak is recognized. We developed an algorithm to help in the management of chyle leak, as represented in Fig. 2.

Of those patients who developed a chyle leak, there were three in-hospital deaths, of whom one patient underwent surgical management and two patients were in the non-surgical group. Causes of death were septic shock following infarcted colonic herniation into the chest (*n* = 1), necrosis of gastric pull-up treated with intercostal muscle flap followed by multi-organ failure (*n* = 1), and hepatorenal failure (*n* = 1). None of these deaths were directly associated with the development of chyle leak.

## Discussion

Chyle leak is a recognized complication following esophagectomy, which has been associated with a high risk of mortality (reaching 50%).^8^,^18^ In the present study, the incidence of chyle leak after Ivor–Lewis esophagectomy was 5%, consistent with previously reported studies. This study describes the clinical and oncological outcomes of patients developing a chyle leak over 20 years and the different strategies adopted from a tertiary care unit. Regarding overall survival, there was no significant difference in perioperative mortality or long-term overall survival between those patients who developed a chyle leak and those who did not; however, chyle leak was associated with a doubling in the length of critical care stay (median: 2 vs. 4 days; *p* = 0.002). While inpatient stay was not noted to be statistically different between groups, the median stays were 15 versus 19 days (*p* = 0.064) and may not have reached statistical significance due to the small number of patients in the chyle leak group. In addition, in contrast to previous studies,^1^–^3^ differences relative to complications, in particular pulmonary complications, were not observed.

Neither tumor characteristics nor employment of neoadjuvant chemotherapy were found to be statistically different between the two groups. The only factor that was found to be prognostic of chyle leak was a slightly lower BMI (median 24 vs. 26; *p* = 0.001).

In addition, aberrant anatomy in the form of a duplex duct system in several female patients, and, on one occasion, a triplex system, has been observed that has not been seen in male patients.^19^ Thoracic duct variations have been largely demonstrated in the literature, including absence of cisterna chyli, left-sided thoracic duct, complete right-sided thoracic duct emptying into the right venous angle, proximal and distal partial duplication, and plexiform variation.^20^ However, other series have found no significant association between sex and incidence of chyle leak.^21^

Regarding surgical technique, en bloc ligation and excision of the thoracic duct was standard practice within this cohort of patients. The aim of this procedure is to reduce the incidence of chyle leak, which is a potential risk with a radical lymphadenectomy. The efficacy of routine ligation has been described by Dougenis et al., who reported a significant decrease in the incidence of chylothorax when routine ligation was undertaken (2.1% vs. 9%),^21^ while Merigliano et al. reported no further cases of chyle leak in 106 patients after instituting this policy.^22^ However, it has been suggested that active attempts to identify and ligate the thoracic duct may in fact lead to its damage.^11^ This was evaluated by Lin et al., who compared routine versus selective ligation of the thoracic duct and used preoperative oral ingestion of olive oil to stimulate chyle production in the group undergoing selective ligation.^23^ They found that selective ligation led to a significantly lower incidence of chyle leak after esophagectomy.

Diagnostic criteria for chyle leak vary between series, with some electing to include only patients with high-volume, biochemically confirmed leaks. The Esophageal Complications Consensus Group (ECCG) categorized chyle leak according to volume and intervention, which graded the level of severity, with only 1% of patients (23% of those who developed a chyle leak) in the most severe group requiring intervention or surgical treatment.^24^,^25^ In the present series, all patients with chyle drainage totaling > 500 mL/24 h were included, as were all patients where there were concerns regarding chylous effluence in the drain that led to a change to using an MCT feed. This may be regarded as a low threshold for diagnosis, but allows identification and appropriate monitoring so that timely treatment of minor and major chyle leaks can be initiated. Furthermore, 40% of patients underwent surgical treatment. This not only reflects an aggressive management strategy for those with high-volume leaks but also demonstrates that the majority of leaks are lower volume and settle with conservative management, the ethos being that prompt intervention may address the problem, with minimal impact on patients and a small increase in hospital stay.

The principles of conservative management of chyle leak include reduction of chyle flow, drainage of the pleural cavity for chylothorax, nutritional support, and the prevention of septic complications.^26^ Immunosuppression is problematic, particularly in those who have a prolonged chyle leak. The opportunistic infection with *Pneumocystis carinii* on a background of massive chylous ascites has been previously reported and prompted the introduction of routine prophylactic antimicrobials in cases of chyle leak.^27^ The two other series detailing rates of infective complications have reported increased rates, particularly rates of respiratory infections in patients with chyle leaks.^1^,^5^ In the present series, the rates of wound and respiratory complications were not significantly raised in patients with chyle leak compared with the other group, however prophylaxis with cotrimoxazole is employed as standard in patients with a chyle leak and low lymphocyte count.

In recent years, there has been a move towards earlier surgical intervention for postoperative chyle leak. Cerfolio suggested reoperation when drainage volumes exceeded 1000 mL/day for 5 days,^2^ while Alexiou et al. suggested reoperation for those with high output chylothorax not settling after a week of conservative management.^5^ For very high-volume leaks (> 2000 mL/day), early surgery after only 2 days of conservative treatment has been proposed,^1^ while Merrigan et al.^26^ advocate reoperation immediately after the diagnosis has been made. An advantage of early reoperation is the opportunity to repair the leak before the patient becomes significantly weakened and immunocompromised by the ongoing loss of chyle. In this study, there were only two deaths among patients undergoing surgical management, compared with eight in the non-surgical group, suggesting that this could be considered a safe and effective treatment. In the majority of our cases (82%), the site of leak was clearly identified and treated at reoperation. Preoperative administration of a cream bolus, lymphoscintigrapy, lymphangiography, and computed tomography (CT) scanning have all been suggested as aids to identify the leak site, but the authors have not found routine use of these techniques to be necessary. Multiple alternative approaches to the management of chyle leak have been described, such as a thoracoscopic or abdominal/transhiatal approach if the chyle leak was originating from the abdomen.^28^–^31^ These have arisen both out of a desire to avoid the morbidity perceived to be associated with re-thoracotomy and to provide alternatives where surgical treatment has failed.

A further option to treat chylothorax is the use of pleuro-peritoneal shunts, which have been demonstrated to be able to provide internal drainage of chylothorax. Chyle can be reabsorbed by the peritoneum preventing the nutritional, fluid, and immunological losses associated with external drainage.^31^ In this series, this has been employed in a small number of patients. Shunts can be a successful option, either as a first-line treatment or in cases resistant to surgical management. However, outcomes with shunts were inferior to those for surgery and the authors advocate surgical intervention as the first-line treatment of choice for high-volume leaks.

In recent years, percutaneous thoracic duct embolization has become the standard of care for high-output chylothoraces.^32^ In cases of chylous output < 1 L/day, conservative management with dietary restriction is a reasonable initial approach, which could be followed by embolization; however, surgery remains an indication in case of failure or success.^20^,^32^,^33^ Unfortunately, this technique is not available at our institution.

There are a number of limitations to this study. While the data for patients within this study, and whether or not there was a chyle leak, was collected at the time of their inpatient stay, not all patients presumed to have a chyle leak had biochemical testing. This could potentially lead to an overestimation of the incidence.

Furthermore, precise data were not available on the length of time that conservative management was employed before progressing to a surgical intervention. In general, most patients who show no response to conservative management and continue to have a high-output chyle leak will have surgery within 24–48 h. While all patients in the cohort had a feeding jejunostomy placed at the time of the original operation, the use of enteral feeding may have had some degree of variability that contributed towards delayed observation of a chylous output and hence the spread of time seen for surgical intervention. In addition, early employment of an MCT feed may lead to some delay in surgery, as time is needed to see if this strategy has a significant impact. The more challenging decision is in those who have some reduction in chyle output with conservative treatment but still have a relatively high output.

## Conclusion

Radical two-field nodal dissection during esophagectomy is associated with an uncommon complication such as chyle leak, even with routine intraoperative identification and ligation of the thoracic duct. Chyle leak appears to have no long-term impact on patient survival. Early use of conservative treatment and, where appropriate, antimicrobial therapy, may address the issue of the many leaks observed; however, where high-volume leaks occur and persist, early surgical intervention is recommended. This study demonstrates that early surgical intervention for major chyle leaks is safe and effective.
